# Supplementary material for: Changes in soil carbon, nitrogen, and phosphorus in Pinus massoniana forest along altitudinal gradients of subtropical karst mountains
Source: PeerJ. 2023 Mar 30;11:e15198. doi: 10.7717/peerj.15198 (PMC10066882; doi:10.7717/peerj.15198)
Supplement: Supplemental Information 1 — Mean DBH: mean diameter at breast high; Mean TH: mean tree’s height. [file peerj-11-15198-s001.docx]

| **Altitude** | **Plot** | **Geographical coordinates of the center point** | **Excat altitude (m)** | **Slope (°)** | **Age of forest (a)** | **Average diameter at breast height of *P. massoniana* (cm)** | **Average height of *P. massoniana* (m)** | **Canopy Density (%)** |
| --- | --- | --- | --- | --- | --- | --- | --- | --- |
| 1200 | Ⅰ-1 | 26°24′41.17″N, 106°54′19.23″E | 1200 | 25 | 41 | 40.00 | 13.63 | 53 |
|  | Ⅰ-2 | 26°24′41.58″N, 106°54′16.91″E | 1202 | 35 | 40 | 34.46 | 13.87 | 81 |
|  | Ⅰ-3 | 26°24′41.58″N, 106°54′16.29″E | 1204 | 30 | 42 | 44.56 | 15.57 | 84 |
| 1300 | Ⅱ-1 | 26°24′27.68″N, 106°53′25.27″E | 1303 | 18 | 53 | 65.71 | 17.25 | 77 |
|  | Ⅱ-2 | 26°24′28.30″N, 106°53′25.66″E | 1308 | 9 | 52 | 62.04 | 16.70 | 72 |
|  | Ⅱ-3 | 26°24′32.14″N, 106°53′27.39″E | 1300 | 32 | 52 | 72.40 | 16.03 | 78 |
| 1400 | Ⅲ-1 | 26°24′10.00″N, 106°53′27.09″E | 1401 | 15 | 50 | 73.14 | 19.00 | 76 |
|  | Ⅲ-2 | 26°24′09.44″N, 106°53′32.42″E | 1409 | 4 | 51 | 53.12 | 19.07 | 74 |
|  | Ⅲ-3 | 26°24′09.44″N, 106°53′29.17″E | 1404 | 6 | 50 | 62.35 | 17.98 | 77 |
| 1500 | Ⅳ-1 | 26°23′41.01″N, 106°53′37.48″E | 1504 | 12 | 52 | 57.08 | 16.02 | 71 |
|  | Ⅳ-2 | 26°23′38.54″N, 106°53′37.08″E | 1506 | 14 | 48 | 56.63 | 15.96 | 72 |
|  | Ⅳ-3 | 26°23′40.14″N, 106°53′40.76″E | 1500 | 7 | 45 | 53.47 | 15.68 | 68 |
| 1600 | Ⅴ-1 | 26°22′24.85″N, 106°54′46.26″E | 1614 | 0 | 9 | 8.39 | 3.05 | \ |
|  | Ⅴ-2 | 26°22′24.85″N, 106°54′46.26″E | 1610 | 0 | 8 | 7.14 | 3.83 | \ |
|  | Ⅴ-3 | 26°22′33.50″N, 106°54′48.81″E | 1617 | 8 | 10 | 6.73 | 3.04 | \ |
