# Supplementary material for: Changes in soil carbon, nitrogen, and phosphorus in Pinus massoniana forest along altitudinal gradients of subtropical karst mountains
Source: PeerJ. 2023 Mar 30;11:e15198. doi: 10.7717/peerj.15198 (PMC10066882; doi:10.7717/peerj.15198)
Supplement: Supplemental Information 8 [file peerj-11-15198-s008.docx]

| **Basic statistics of soil properties** | | | | | | |
| --- | --- | --- | --- | --- | --- | --- |
| **altitude** | **soil layer** | **soil properties** | **degrees of freedom** | **mean** | **standard error** | **letter** |
| 1200 | topsoil | SOC | 9 | 23.4804537 | 0.30060362 | CB a |
| 1300 | topsoil | SOC | 9 | 20.29524253 | 0.432989173 | CD a |
| 1400 | topsoil | SOC | 9 | 30.36486137 | 0.4647043 | BA a |
| 1500 | topsoil | SOC | 9 | 36.97239404 | 1.553429352 | A a |
| 1600 | topsoil | SOC | 9 | 12.38295662 | 1.065037076 | D a |
| 1200 | subsoil | SOC | 9 | 6.62185047 | 0.83563077 | B b |
| 1300 | subsoil | SOC | 9 | 8.893453209 | 0.906308056 | B b |
| 1400 | subsoil | SOC | 3 | 13.68453261 | 0.237521446 | AB b |
| 1500 | subsoil | SOC | 9 | 17.17761988 | 0.774247637 | A b |
| 1600 | subsoil | SOC | 9 | 5.829571535 | 1.018901344 | B b |
| 1200 | topsoil | TP | 9 | 0.21261696 | 0.00822939 | BA a |
| 1300 | topsoil | TP | 9 | 0.1417216 | 0.009277827 | CD a |
| 1400 | topsoil | TP | 9 | 0.192720782 | 0.012553736 | BC a |
| 1500 | topsoil | TP | 9 | 0.26609752 | 0.018520112 | A a |
| 1600 | topsoil | TP | 9 | 0.115657004 | 0.00686589 | D a |
| 1200 | subsoil | TP | 9 | 0.173555484 | 0.004469257 | AB b |
| 1300 | subsoil | TP | 9 | 0.095432533 | 0.004427374 | C b |
| 1400 | subsoil | TP | 3 | 0.102497707 | 0.002399444 | BC b |
| 1500 | subsoil | TP | 9 | 0.190546987 | 0.003076453 | A b |
| 1600 | subsoil | TP | 9 | 0.099143227 | 0.009214388 | C a |
| 1200 | topsoil | AP | 9 | 1.077396667 | 0.121684442 | A a |
| 1300 | topsoil | AP | 9 | 1.006113333 | 0.02657928 | A a |
| 1400 | topsoil | AP | 9 | 1.1609 | 0.146862591 | A a |
| 1500 | topsoil | AP | 9 | 0.798373333 | 0.037492096 | A a |
| 1600 | topsoil | AP | 9 | 1.06314 | 0.178893663 | A a |
| 1200 | subsoil | AP | 9 | 0.550918333 | 0.058174381 | A b |
| 1300 | subsoil | AP | 9 | 0.432791667 | 0.034522101 | A b |
| 1400 | subsoil | AP | 3 | 0.644605 | 0.026101931 | A b |
| 1500 | subsoil | AP | 9 | 0.460286667 | 0.038211254 | A b |

| 1600 | subsoil | AP | 9 | 0.624238333 | 0.106623925 | A ab |
| --- | --- | --- | --- | --- | --- | --- |
| 1200 | topsoil | TN | 9 | 1.638268152 | 0.028052109 | A a |
| 1300 | topsoil | TN | 9 | 1.130088588 | 0.017953594 | B a |
| 1400 | topsoil | TN | 9 | 1.760717914 | 0.067380599 | A a |
| 1500 | topsoil | TN | 9 | 2.062168861 | 0.104957611 | A a |
| 1600 | topsoil | TN | 9 | 0.877721979 | 0.059027311 | B a |
| 1200 | subsoil | TN | 9 | 0.500660261 | 0.054377929 | BC b |
| 1300 | subsoil | TN | 9 | 0.511312607 | 0.03740806 | BC b |
| 1400 | subsoil | TN | 3 | 0.712453964 | 0.004883931 | BA b |
| 1500 | subsoil | TN | 9 | 1.043251081 | 0.011092876 | A b |
| 1600 | subsoil | TN | 9 | 0.41152519 | 0.050931465 | C b |
| 1200 | topsoil | AN | 9 | 134.9218572 | 4.433785996 | A a |

| 1300 | topsoil | AN | 9 | 96.29503519 | 2.293397486 | B a |
| --- | --- | --- | --- | --- | --- | --- |
| 1400 | topsoil | AN | 9 | 145.6083522 | 11.45260763 | A a |
| 1500 | topsoil | AN | 9 | 162.8621842 | 6.72535955 | A a |
| 1600 | topsoil | AN | 9 | 69.75366754 | 3.96490854 | B a |
| 1200 | subsoil | AN | 9 | 49.50761696 | 2.60622748 | B b |
| 1300 | subsoil | AN | 9 | 51.26926948 | 5.463172326 | B b |
| 1400 | subsoil | AN | 3 | 61.20447152 | 4.962527718 | AB b |
| 1500 | subsoil | AN | 9 | 88.61371209 | 2.940512289 | A b |

| 1600 | subsoil | AN | 9 | 37.96620233 | 1.787242347 | B b |
| --- | --- | --- | --- | --- | --- | --- |

Note: The different capital letters indicate that the same soil property of different altitudes in same soil layer are significantly different on 0.05 levels, and different small letters indicate that the same soil property of different layer in same altitude are significantly different on 0.05 levels.

| **The exact p-value between two elevations in the same soil layer** |
| --- |

| **soil layer** | **soil properties** | **Comparison of different altitudes** | **exact p-value** |
| --- | --- | --- | --- |
| topsoil | SOC | 1200-1300 | 0.167019 |
| topsoil | SOC | 1200-1400 | 0.170437 |
| topsoil | SOC | 1300-1400 | 0.006812 |
| topsoil | SOC | 1200-1500 | 0.008122 |
| topsoil | SOC | 1300-1500 | 0.000051 |
| topsoil | SOC | 1400-1500 | 0.162276 |
| topsoil | SOC | 1200-1600 | 0.008174 |
| topsoil | SOC | 1300-1600 | 0.194785 |
| topsoil | SOC | 1400-1600 | 0.000065 |
| topsoil | SOC | 1500-1600 | 0.000000 |
| topsoil | TP | 1200-1300 | 0.007222 |
| topsoil | TP | 1200-1400 | 0.369393 |
| topsoil | TP | 1300-1400 | 0.063449 |
| topsoil | TP | 1200-1500 | 0.269459 |
| topsoil | TP | 1300-1500 | 0.000123 |
| topsoil | TP | 1400-1500 | 0.045455 |
| topsoil | TP | 1200-1600 | 0.000836 |
| topsoil | TP | 1300-1600 | 0.495228 |
| topsoil | TP | 1400-1600 | 0.014198 |
| topsoil | TP | 1500-1600 | 0.000010 |
| topsoil | AP | 1200-1300 | 0.676500 |
| topsoil | AP | 1200-1400 | 0.598724 |
| topsoil | AP | 1300-1400 | 0.767034 |
| topsoil | AP | 1200-1500 | 0.304855 |
| topsoil | AP | 1300-1500 | 0.139219 |
| topsoil | AP | 1400-1500 | 0.125696 |
| topsoil | AP | 1200-1600 | 0.563481 |
| topsoil | AP | 1300-1600 | 0.411153 |

| topsoil | AP | 1400-1600 | 0.295673 |
| --- | --- | --- | --- |
| topsoil | AP | 1500-1600 | 0.584135 |
| topsoil | TN | 1200-1300 | 0.047558 |
| topsoil | TN | 1200-1400 | 0.350624 |
| topsoil | TN | 1300-1400 | 0.003578 |
| topsoil | TN | 1200-1500 | 0.088479 |
| topsoil | TN | 1300-1500 | 0.000166 |
| topsoil | TN | 1400-1500 | 0.389582 |
| topsoil | TN | 1200-1600 | 0.003284 |
| topsoil | TN | 1300-1600 | 0.382809 |
| topsoil | TN | 1400-1600 | 0.000169 |
| topsoil | TN | 1500-1600 | 0.000004 |
| topsoil | AN | 1200-1300 | 0.036001 |
| topsoil | AN | 1200-1400 | 0.660139 |
| topsoil | AN | 1300-1400 | 0.012399 |
| topsoil | AN | 1200-1500 | 0.205298 |
| topsoil | AN | 1300-1500 | 0.000564 |
| topsoil | AN | 1400-1500 | 0.379428 |
| topsoil | AN | 1200-1600 | 0.000587 |
| topsoil | AN | 1300-1600 | 0.208584 |
| topsoil | AN | 1400-1600 | 0.000139 |
| topsoil | AN | 1500-1600 | 0.000003 |
| subsoil | SOC | 1200-1300 | 0.493688 |
| subsoil | SOC | 1200-1400 | 0.081400 |
| subsoil | SOC | 1300-1400 | 0.220244 |
| subsoil | SOC | 1200-1500 | 0.000277 |
| subsoil | SOC | 1300-1500 | 0.003631 |
| subsoil | SOC | 1400-1500 | 0.526739 |
| subsoil | SOC | 1200-1600 | 0.836217 |
| subsoil | SOC | 1300-1600 | 0.473175 |
| subsoil | SOC | 1400-1600 | 0.070812 |
| subsoil | SOC | 1500-1600 | 0.000225 |
| subsoil | TP | 1200-1300 | 0.002909 |
| subsoil | TP | 1200-1400 | 0.095616 |
| subsoil | TP | 1300-1400 | 0.813013 |
| subsoil | TP | 1200-1500 | 0.364995 |
| subsoil | TP | 1300-1500 | 0.000080 |
| subsoil | TP | 1400-1500 | 0.013680 |
| subsoil | TP | 1200-1600 | 0.008889 |
| subsoil | TP | 1300-1600 | 0.754719 |
| subsoil | TP | 1400-1600 | 0.872243 |
| subsoil | TP | 1500-1600 | 0.000254 |
| subsoil | AP | 1200-1300 | 0.428078 |
| subsoil | AP | 1200-1400 | 0.441958 |
| subsoil | AP | 1300-1400 | 0.364750 |
| subsoil | AP | 1200-1500 | 0.452501 |
| subsoil | AP | 1300-1500 | 0.840391 |
| subsoil | AP | 1400-1500 | 0.305899 |
| subsoil | AP | 1200-1600 | 0.795973 |
| subsoil | AP | 1300-1600 | 0.517564 |
| subsoil | AP | 1400-1600 | 0.383971 |
| subsoil | AP | 1500-1600 | 0.426681 |
| subsoil | TN | 1200-1300 | 0.772175 |
| subsoil | TN | 1200-1400 | 0.156754 |
| subsoil | TN | 1300-1400 | 0.120454 |
| subsoil | TN | 1200-1500 | 0.001642 |
| subsoil | TN | 1300-1500 | 0.000802 |
| subsoil | TN | 1400-1500 | 0.537149 |
| subsoil | TN | 1200-1600 | 0.465618 |
| subsoil | TN | 1300-1600 | 0.542700 |
| subsoil | TN | 1400-1600 | 0.044589 |
| subsoil | TN | 1500-1600 | 0.000079 |
| subsoil | AN | 1200-1300 | 0.679140 |
| subsoil | AN | 1200-1400 | 0.567198 |
| subsoil | AN | 1300-1400 | 0.427273 |
| subsoil | AN | 1200-1500 | 0.009665 |
| subsoil | AN | 1300-1500 | 0.003471 |
| subsoil | AN | 1400-1500 | 0.210959 |
| subsoil | AN | 1200-1600 | 0.077245 |
| subsoil | AN | 1300-1600 | 0.163360 |
| subsoil | AN | 1400-1600 | 0.084905 |
| subsoil | AN | 1500-1600 | 0.000005 |

| **The exact p-value between two elevations in the same altitude** | | | |
| --- | --- | --- | --- |
| **altitude** | **soil properties** | **Comparison of different soil layer** | **exact p-value** |
| 1200 | SOC | topsoil-subsoil | 2.10E-07 |
| 1200 | TP | topsoil-subsoil | 2.09E-07 |
| 1200 | AP | topsoil-subsoil | 1.54E-05 |
| 1200 | TN | topsoil-subsoil | 0.00967176 |
| 1200 | AN | topsoil-subsoil | 2.08E-07 |
| 1300 | SOC | topsoil-subsoil | 3.98E-06 |
| 1300 | TP | topsoil-subsoil | 2.09E-07 |
| 1300 | AP | topsoil-subsoil | 0.000180987 |
| 1300 | TN | topsoil-subsoil | 1.53E-05 |
| 1300 | AN | topsoil-subsoil | 2.09E-07 |
| 1400 | SOC | topsoil-subsoil | 3.98E-06 |
| 1400 | TP | topsoil-subsoil | 2.09E-07 |
| 1400 | AP | topsoil-subsoil | 0.000180987 |
| 1400 | TN | topsoil-subsoil | 1.53E-05 |
| 1400 | AN | topsoil-subsoil | 2.09E-07 |
| 1500 | SOC | topsoil-subsoil | 3.98E-06 |
| 1500 | TP | topsoil-subsoil | 2.09E-07 |
| 1500 | AP | topsoil-subsoil | 0.000180987 |
| 1500 | TN | topsoil-subsoil | 1.53E-05 |
| 1500 | AN | topsoil-subsoil | 2.09E-07 |
| 1600 | SOC | topsoil-subsoil | 3.98E-06 |
| 1600 | TP | topsoil-subsoil | 2.09E-07 |
| 1600 | AP | topsoil-subsoil | 0.000180987 |
| 1600 | TN | topsoil-subsoil | 1.53E-05 |
