# Supplementary material for: Changes in soil carbon, nitrogen, and phosphorus in Pinus massoniana forest along altitudinal gradients of subtropical karst mountains
Source: PeerJ. 2023 Mar 30;11:e15198. doi: 10.7717/peerj.15198 (PMC10066882; doi:10.7717/peerj.15198)
Supplement: Supplemental Information 9 [file peerj-11-15198-s009.docx]

| **soil layer** | **soil properties** | **degrees of freedom** | **R2** | **exact p-value** | **fitted equation** |
| --- | --- | --- | --- | --- | --- |
| topsoil | SOC | 45 | 0.402 | < 0.0001 | y = - 0.000331x^2^ + 0.92x - 609 |
| topsoil | TN | 45 | 0.218 | 0.00579 | y =- 1.2e-05 x^2^ + 0.033x - 21 |
| topsoil | AN | 45 | 0.243 | 0.00289 | y = - 0.00101x^2^ + 2.76x - 1743 |
| topsoil | TP | 45 | 0.0924 | 0.131 | y = - 9.77e-07x^2^ + 0.00266x - 1.61 |
| topsoil | AP | 45 | 0.0114 | 0.786 | y = 1.11e-06x^2^ - 0.00333x + 3.5 |
| subsoil | SOC | 39 | 0.496 | <0.0001 | y = - 0.000216x^2^ + 0.61x - 417 |
| subsoil | TN | 39 | 0.360 | 0.000321 | y = - 9.5e-06x^2^+ 0.0269 x -18.3 |
| subsoil | AN | 39 | 0.342 | 0.000542 | y = - 0.00075x^2^ + 2.12x - 1416 |
| subsoil | TP | 39 | 0.0334 | 0.542 | y = 7.94e-08x^2^ - 0.000276x + 0.366 |
| subsoil | AP | 39 | 0.0758 | 0.242 | y = 0.00000298x^2^ - 0.00816x + 6.05 |
