# Supplementary material for: Changes in soil carbon, nitrogen, and phosphorus in Pinus massoniana forest along altitudinal gradients of subtropical karst mountains
Source: PeerJ. 2023 Mar 30;11:e15198. doi: 10.7717/peerj.15198 (PMC10066882; doi:10.7717/peerj.15198)
Supplement: Supplemental Information 10 [file peerj-11-15198-s010.docx]

matrix of correlations in topsoil

|  | SOC | TN | AN | TP | AP | Alt | Slp | pH | SBD | SMC | Clay | Silt | Sand | TG | EEG |
| --- | --- | --- | --- | --- | --- | --- | --- | --- | --- | --- | --- | --- | --- | --- | --- |
| SOC | 1 |  |  |  |  |  |  |  |  |  |  |  |  |  |  |
| TN | 0.874415 | 1 |  |  |  |  |  |  |  |  |  |  |  |  |  |
| AN | 0.824390 | 0.935653 | 1 |  |  |  |  |  |  |  |  |  |  |  |  |
| TP | 0.788023 | 0.862014 | 0.843744 | 1 |  |  |  |  |  |  |  |  |  |  |  |
| AP | -0.174724 | 0.044299 | 0.084009 | 0.029332 | 1 |  |  |  |  |  |  |  |  |  |  |
| Alt | -0.095584 | -0.159741 | -0.257134 | -0.209337 | -0.249973 | 1 |  |  |  |  |  |  |  |  |  |
| Slp | 0.116144 | 0.172862 | 0.179584 | 0.243906 | 0.271962 | -0.786287 | 1 |  |  |  |  |  |  |  |  |
| pH | -0.002834 | 0.245220 | 0.261578 | 0.473285 | 0.075543 | -0.358955 | 0.397919 | 1 |  |  |  |  |  |  |  |
| SBD | -0.587088 | -0.452988 | -0.442929 | -0.437593 | 0.248674 | 0.125832 | -0.210684 | 0.053325 | 1 |  |  |  |  |  |  |
| SMC | 0.566930 | 0.456678 | 0.466976 | 0.488586 | -0.228703 | -0.135511 | 0.248341 | 0.027553 | -0.966798 | 1 |  |  |  |  |  |
| Clay | 0.318564 | 0.406735 | 0.433052 | 0.438851 | 0.251879 | -0.802311 | 0.644644 | 0.533144 | -0.114248 | 0.112799 | 1 |  |  |  |  |
| Silt | 0.429922 | 0.481402 | 0.532020 | 0.551917 | 0.179311 | -0.791316 | 0.640991 | 0.504103 | -0.187358 | 0.190191 | 0.932529 | 1 |  |  |  |
| Sand | -0.395652 | -0.473348 | -0.522450 | -0.531344 | -0.195090 | 0.810649 | -0.657618 | -0.533188 | 0.150856 | -0.155072 | -0.956811 | -0.992193 | 1 |  |  |
| TG | 0.655599 | 0.508203 | 0.560925 | 0.381724 | -0.122195 | -0.298851 | -0.002378 | -0.258981 | -0.431752 | 0.353623 | 0.245429 | 0.328996 | -0.299868 | 1 |  |
| EEG | 0.539807 | 0.443844 | 0.478126 | 0.271775 | 0.002603 | -0.111922 | -0.124868 | -0.325434 | -0.111598 | 0.038209 | 0.074619 | 0.197510 | -0.160216 | 0.705689 | 1 |

asymptotic P-values in topsoil

|  | SOC | TN | AN | TP | AP | Alt | Slp | pH | SBD | SMC | Clay | Silt | Sand | TG | EEG |
| --- | --- | --- | --- | --- | --- | --- | --- | --- | --- | --- | --- | --- | --- | --- | --- |
| SOC | NA |  |  |  |  |  |  |  |  |  |  |  |  |  |  |
| TN | 0.000000 | NA |  |  |  |  |  |  |  |  |  |  |  |  |  |
| AN | 0.000000 | 0.000000 | NA |  |  |  |  |  |  |  |  |  |  |  |  |
| TP | 0.000000 | 0.000000 | 0.000000 | NA |  |  |  |  |  |  |  |  |  |  |  |
| AP | 0.250986 | 0.772620 | 0.583237 | 0.848313 | NA |  |  |  |  |  |  |  |  |  |  |
| Alt | 0.532243 | 0.294559 | 0.088162 | 0.167553 | 0.097704 | NA |  |  |  |  |  |  |  |  |  |
| Slp | 0.447394 | 0.256150 | 0.237835 | 0.106393 | 0.070720 | 0.000000 | NA |  |  |  |  |  |  |  |  |
| pH | 0.985257 | 0.104462 | 0.082617 | 0.001025 | 0.621868 | 0.015457 | 0.006790 | NA |  |  |  |  |  |  |  |
| SBD | 0.000022 | 0.001780 | 0.002312 | 0.002647 | 0.099517 | 0.410147 | 0.164776 | 0.727916 | NA |  |  |  |  |  |  |
| SMC | 0.000049 | 0.001614 | 0.001221 | 0.000661 | 0.130757 | 0.374777 | 0.099985 | 0.857419 | 0.000000 | NA |  |  |  |  |  |
| Clay | 0.032944 | 0.005560 | 0.002965 | 0.002564 | 0.095090 | 0.000000 | 0.000002 | 0.000163 | 0.454892 | 0.460671 | NA |  |  |  |  |
| Silt | 0.003204 | 0.000814 | 0.000169 | 0.000085 | 0.238563 | 0.000000 | 0.000002 | 0.000415 | 0.217794 | 0.210790 | 0.000000 | NA |  |  |  |
| Sand | 0.007141 | 0.001023 | 0.000232 | 0.000173 | 0.199052 | 0.000000 | 0.000001 | 0.000163 | 0.322578 | 0.309080 | 0.000000 | 0.000000 | NA |  |  |
| TG | 0.000001 | 0.000365 | 0.000061 | 0.009670 | 0.423917 | 0.046133 | 0.987629 | 0.085823 | 0.003062 | 0.017173 | 0.104158 | 0.027335 | 0.045361 | NA |  |
| EEG | 0.000130 | 0.002258 | 0.000894 | 0.070921 | 0.986458 | 0.464187 | 0.413770 | 0.029153 | 0.465488 | 0.803210 | 0.626147 | 0.193428 | 0.293108 | 0.000000 | NA |

matrix of correlations in subsoil

|  | SOC | TN | AN | TP | AP | Alt | Slp | pH | SBD | SWC | Clay | Silt | Sand | EEG | TG |
| --- | --- | --- | --- | --- | --- | --- | --- | --- | --- | --- | --- | --- | --- | --- | --- |
| SOC | 1 | 0.902182 | 0.744304 | 0.447234 | 0.002735 | 0.165655 | -0.209758 | -0.191795 | -0.581305 | 0.592439 | -0.055474 | -0.097070 | 0.084422 | 0.665317 | 0.602966 |
| TN |  | 1 | 0.765537 | 0.584051 | 0.149848 | 0.079431 | -0.174453 | -0.043621 | -0.610976 | 0.642770 | 0.037367 | 0.060045 | -0.069364 | 0.557817 | 0.561260 |
| AN |  |  | 1 | 0.600233 | -0.189726 | -0.107159 | -0.005795 | -0.348077 | -0.570807 | 0.623462 | 0.112866 | 0.136702 | -0.143595 | 0.483824 | 0.678953 |
| TP |  |  |  | 1 | 0.187430 | -0.170023 | 0.230806 | 0.025932 | -0.422209 | 0.497621 | 0.293683 | 0.364612 | -0.375664 | 0.339508 | 0.338698 |
| AP |  |  |  |  | 1 | -0.020251 | 0.092888 | 0.103172 | 0.110593 | -0.104314 | 0.176491 | 0.216224 | -0.216944 | 0.026332 | -0.041726 |
| Alt |  |  |  |  |  | 1 | -0.872205 | 0.051412 | 0.160042 | -0.171251 | -0.779117 | -0.853143 | 0.848204 | -0.123301 | -0.351844 |
| Slp |  |  |  |  |  |  | 1 | -0.081304 | -0.111885 | 0.108227 | 0.814987 | 0.815514 | -0.813726 | 0.043596 | 0.177431 |
| pH |  |  |  |  |  |  |  | 1 | -0.022688 | 0.083156 | 0.151388 | 0.134610 | -0.134110 | -0.149600 | -0.315608 |
| SBD |  |  |  |  |  |  |  |  | 1 | -0.969231 | -0.315518 | -0.233198 | 0.237360 | -0.203239 | -0.572672 |
| SWC |  |  |  |  |  |  |  |  |  | 1 | 0.322300 | 0.259717 | -0.264487 | 0.212753 | 0.564777 |
| Clay |  |  |  |  |  |  |  |  |  |  | 1 | 0.940277 | -0.943058 | 0.150420 | 0.316226 |
| Silt |  |  |  |  |  |  |  |  |  |  |  | 1 | -0.999140 | 0.200607 | 0.366397 |
| Sand |  |  |  |  |  |  |  |  |  |  |  |  | 1 | -0.209019 | -0.363682 |
| EEG |  |  |  |  |  |  |  |  |  |  |  |  |  | 1 | 0.610324 |
| TG |  |  |  |  |  |  |  |  |  |  |  |  |  |  | 1 |

asymptotic P-values in subsoil

|  | SOC | TN | AN | TP | AP | Alt | Slp | pH | SBD | SWC | Clay | Silt | Sand | EEG | TG |
| --- | --- | --- | --- | --- | --- | --- | --- | --- | --- | --- | --- | --- | --- | --- | --- |
| SOC | NA | 0.000000 | 0.000000 | 0.004310 | 0.986818 | 0.313530 | 0.199972 | 0.242126 | 0.000104 | 0.000071 | 0.737307 | 0.556621 | 0.609370 | 0.000004 | 0.000049 |
| TN |  | NA | 0.000000 | 0.000095 | 0.362554 | 0.630755 | 0.288151 | 0.792028 | 0.000036 | 0.000010 | 0.821323 | 0.716526 | 0.674787 | 0.000225 | 0.000201 |
| AN |  |  | NA | 0.000054 | 0.247341 | 0.516140 | 0.972069 | 0.029899 | 0.000148 | 0.000022 | 0.493906 | 0.406633 | 0.383150 | 0.001805 | 0.000002 |
| TP |  |  |  | NA | 0.253213 | 0.300759 | 0.157462 | 0.875480 | 0.007420 | 0.001267 | 0.069581 | 0.022483 | 0.018436 | 0.034472 | 0.034933 |
| AP |  |  |  |  | NA | 0.902609 | 0.573824 | 0.531959 | 0.502701 | 0.527403 | 0.282467 | 0.186138 | 0.184641 | 0.873575 | 0.800880 |
| Alt |  |  |  |  |  | NA | 0.000000 | 0.755933 | 0.330435 | 0.297229 | 0.000000 | 0.000000 | 0.000000 | 0.454567 | 0.028053 |
| Slp |  |  |  |  |  |  | NA | 0.622691 | 0.497692 | 0.511941 | 0.000000 | 0.000000 | 0.000000 | 0.792148 | 0.279870 |
| pH |  |  |  |  |  |  |  | NA | 0.890956 | 0.614763 | 0.357586 | 0.413920 | 0.415671 | 0.363358 | 0.050325 |
| SBD |  |  |  |  |  |  |  |  | NA | 0.000000 | 0.050394 | 0.153083 | 0.145677 | 0.214631 | 0.000139 |
| SWC |  |  |  |  |  |  |  |  |  | NA | 0.045389 | 0.110334 | 0.103715 | 0.193477 | 0.000180 |
| Clay |  |  |  |  |  |  |  |  |  |  | NA | 0.000000 | 0.000000 | 0.360703 | 0.049852 |
| Silt |  |  |  |  |  |  |  |  |  |  |  | NA | 0.000000 | 0.220754 | 0.021783 |
| Sand |  |  |  |  |  |  |  |  |  |  |  |  | NA | 0.201598 | 0.022854 |
| EEG |  |  |  |  |  |  |  |  |  |  |  |  |  | NA | 0.000037 |
| TG |  |  |  |  |  |  |  |  |  |  |  |  |  |  | NA |
